# Predictors of primary care referrals to a vascular disease prevention lifestyle program among participants in a cluster randomised trial

**DOI:** 10.1186/1472-6963-12-234

**Published:** 2012-08-03

**Authors:** Megan E Passey, Rachel A Laws, Upali W Jayasinghe, Mahnaz Fanaian, Suzanne McKenzie, Gawaine Powell-Davies, David Lyle, Mark F Harris

**Affiliations:** 1 University Centre for Rural Health – North Coast, School of Public Health, University of Sydney, PO Box 3074, Lismore, NSW 2480, Australia; 2 Prevention Research Collaboration, University of Sydney, Sydney, NSW, 2006, Australia; 3 Centre for Primary Health Care and Equity, Faculty of Medicine, University of New South Wales, Sydney, NSW, 2006, Australia; 4 School of Medicine and Dentistry, James Cook University, Townsville, Qld, 4811, Australia; 5 Broken Hill University Department of Rural Health, School of Public Health, University of Sydney, Broken Hill, NSW, 2880, Australia

**Keywords:** Preventive health care, General practice, Health behaviour, Lifestyle modification, Referral

## Abstract

**Background:**

Cardiovascular disease accounts for a large burden of disease, but is amenable to prevention through lifestyle modification. This paper examines patient and practice predictors of referral to a lifestyle modification program (LMP) offered as part of a cluster randomised controlled trial (RCT) of prevention of vascular disease in primary care.

**Methods:**

Data from the intervention arm of a cluster RCT which recruited 36 practices through two rural and three urban primary care organisations were used. In each practice, 160 eligible high risk patients were invited to participate. Practices were randomly allocated to intervention or control groups. Intervention practice staff were trained in screening, motivational interviewing and counselling and encouraged to refer high risk patients to a LMP involving individual and group sessions. Data include patient surveys; clinical audit; practice survey on capacity for preventive care; referral records from the LMP. Predictors of referral were examined using multi-level logistic regression modelling after adjustment for confounding factors.

**Results:**

Of 301 eligible patients, 190 (63.1%) were referred to the LMP. Independent predictors of referral were baseline BMI ≥ 25 (OR 2.87 95%CI:1.10, 7.47), physical inactivity (OR 2.90 95%CI:1.36,6.14), contemplation/preparation/action stage of change for physical activity (OR 2.75 95%CI:1.07, 7.03), rural location (OR 12.50 95%CI:1.43, 109.7) and smaller practice size (1–3 GPs) (OR 16.05 95%CI:2.74, 94.24).

**Conclusions:**

Providing a well-structured evidence-based lifestyle intervention, free of charge to patients, with coordination and support for referral processes resulted in over 60% of participating high risk patients being referred for disease prevention. Contrary to expectations, referrals were more frequent from rural and smaller practices suggesting that these practices may be more ready to engage with these programs.

**Trial registration:**

ACTRN12607000423415

## Background

Cardiovascular disease (CVD) and diabetes together account for 23% of the overall burden of disease in Australia[1]. These conditions share common behavioural and physiological risks factors of which more than half the Australian adult population have at least two[2]. These risk factors are amenable to behavioural interventions and are commonly managed in general practice and the community [3-7]. Recent evidence has demonstrated the need to supplement usual clinical practice with more intensive behavioural interventions, in order to achieve reductions in weight[8,9].

To improve risk factor management, the Australian government introduced preventive health checks for those aged 40–49 years and for at-risk and vulnerable population groups. These were linked to establishment of referral providers for more intensive lifestyle interventions [10]. However their uptake has been disappointingly low[11,12].

In previous research we have reported very low rates of referral from general practice to other services for preventive interventions[13]. These referrals were associated with patients being overweight but not with other risk factors. Barriers to referral include linkages to referral services, their availability, cost to patients and perceived effectiveness [14-17]. The Health Improvement and Prevention Study (HIPS) lifestyle intervention was specifically designed to address some of these by providing an evidence-based program, involving identification of high risk patients through a health assessment, coordination of referral and a service which incurred no direct costs to patients[18].

This paper reports a secondary analysis of data from a a cluster randomised controlled trial (RCT) of prevention of CVD in primary care[18]. It examines patient and practice predictors of referral to the HIPS lifestyle modification program (LMP) offered in the intervention arm of the trial.

## Methods

HIPS was a cluster RCT that aimed to evaluate the impact of a general practice intervention for patients at high risk of vascular disease on changes in behavioural and physiological risk factors. The detailed protocol for the HIPS study has been published elsewhere[18]. Trial registration: ACTRN12607000423415.

### Recruitment

The study was conducted in two rural and three urban Divisions of General Practice (primary care organisations) in New South Wales, Australia. Thirty six practices were invited and 30 agreed to participate. Sixteen practices were randomly allocated to the intervention group.

Patients were eligible for the study if they had attended the practice in the previous 12 months, were aged 40–55 with hypertension and/or hyperlipidemia, or aged 56–65 years. Up to 160 eligible patients were identified by record audit in each practice and were invited to participate by mail. They were excluded if they had pre-existing diabetes, CVD, current severe illness or were unable to speak English.

### Intervention

In intervention practices, general practitioners (GPs) and practice nurses (PNs) were offered a three hour training session in screening, lifestyle counselling, assessment of stage of change and motivational interviewing as well as practice visits and patient educational resources. Participating patients were invited to attend for a health check during which the GP and PN provided brief lifestyle counselling based on the 5As model (ask, assess, advise, assist, and arrange)[19]. Providers were encouraged to refer high risk patients (defined as one or more of: history of gestational diabetes, impaired glucose tolerance or impaired fasting glycaemia, hypertension, hyperlipidaemia, body mass index (BMI) ≥25 or waist circumference >102 cm in males or 88 cm in females, current smoker) to a LMP which was organised by a coordinator from the Division of General Practice. The LMP included an initial visit with a dietician or exercise physiologist for an assessment and individual goal setting, followed by a group education program, adapted from the group component of the “*Counterweight Program – CHANGE*” [20]. The group program, CHANGE for HIPS consisted of four sessions of 1.5 hours over three months, followed by two further sessions at six and nine months. The sessions included both education and physical activity components and used self-management strategies (goal setting, self monitoring, developing practical skills and problem solving) to promote positive dietary and physical activity changes and weight loss.

### Data collection

This paper draws on four sources of data collected as part of the study:

1) Patient survey data

2) Patient clinical audit data

3) Practice questionnaire on capacity for preventive care

4) Lifestyle modification program referral records

#### Patient survey data

Patients completed a mailed survey at baseline and 12 months which was based on the NSW Health Survey[21] and previous research [22,23]. It included questions about: (a) demographic variables (gender, age, postcode of residence, education level, employment status, accommodation type and language spoken at home); (b) self-reported fruit and vegetable intake, with an explanation of what constituted a portion[21], smoking , physical activity [22] and alcohol intake using questions from a widely validated tool, the AUDIT-C, with pictorial representations of what constituted a ‘standard drink’ [23,24], and attempts to change these; (c) the Kessler Psychological Distress Scale (K-10) [25], a ten item questionnaire measuring negative emotional states in the preceding four weeks; (d) readiness for behaviour change (stage of change) for each lifestyle risk factor [26,27]; (e) reported assessment and management of lifestyle risk factors (smoking, nutrition, alcohol and physical activity). Stage of change was assessed by presenting a description of each stage, and asking the respondents to indicate which stage they were in for each of the lifestyle changes [10]. The 12 month survey also included the Porter Novelli’s 10-item scale[28] which categorizes individuals into four distinct groups based on differences in degree of engagement in health enhancement (active versus passive) and degree of independence in health decision making (independent versus doctor dependent).

#### Patient clinical audit data

GPs and PNs were requested to record patient weight, waist circumference and blood pressure at baseline. These data were extracted from patient records by trained data collectors. Results of patients’ baseline fasting serum lipids (total cholesterol, HDL, LDL, triglycerides) and glucose were sent directly to the study centre as well as to their GP by the pathology company.

#### Practice questionnaire on capacity for preventive care

The practice manager or principal general practitioner was asked to complete a questionnaire on practice capacity for preventive care. The questionnaire included questions on practice characteristics (practice location, practice size, employment of practice nurses), the use of written preventive care protocols and linkages between the practice and support services [10].

#### Lifestyle modification program referral records

The program coordinators monitored and recorded GP referrals to the LMP (the outcome measure).

### Data management and analysis

The postcode of each patient’s residence was classified according to the 2006 Index of Relative Socio-economic Advantage and Disadvantage of the Socio-Economic Indexes for Areas (SEIFA) [29]. This was categorised into quintiles. Dichotomous patient characteristics and behavioural risk variables were computed. For stage of change data, contemplation, preparation and action stages were grouped together as these stages were the main target for referral. The following risk classifications were used:

 • Blood pressure ≥ 140/90 on two occasions or on treatment for high blood pressure;

 • Lipids – Total Cholesterol (TC) > 4.5 mmol/L or Low Density Lipoprotein (LDL) > 2.5 mmol/L or Triglyceride (TG) > 2.0 mmol/L or on treatment for it

 • BMI was calculated as body weight in kilograms divided by the square of the reported height in meters, with a BMI of ≥ 25 indicated overweight or obese [30];

 • Reported number of daily portions of fruit and vegetables consumed was summed, with a score of less than seven (five portions of vegetables and two of fruit) indicating inadequate diet, consistent with Australian national guidelines [30];

 • Physical activity scores were calculated using the reported frequency of physical activity per week (scored from 0 to 8) with a score of less than 4 considered inadequate activity levels in accordance with Australian guidelines [31];

 • At-risk alcohol intake was defined as reporting more than two standard drinks consumed on a typical day when drinking, consistent with Australian national guidelines[32];

 • Smokers included those who indicated that they were currently smoking tobacco.

The analysis for this paper includes intervention patients only. Univariate analysis was undertaken using SPSS statistical software (version 17; SPSS, Chicago, IL, USA). Initial univariate analysis compared demographic characteristics, patient health risk profile, health seeking behaviour, readiness to change, and previous GP intervention or referral for diet and physical activity for those referred to the LMP compared to those not referred. The characteristics of the practices which patients attended were also compared. Significant differences for those referred compared to those not referred were examined using the Pearson chi-square test and, if any of the expected frequencies was less than five, the Fisher exact test was used.

Variables found to be significant (P < 0.05) or of borderline significance (P = 0.071 for rural location) in the univariate analysis were entered into a multi-level logistic regression analysis to examine patient and practice factors associated with referral levels. The multi-level analysis was considered appropriate as patient data for referral was highly clustered by practice (ICC = 0.263, equivalent to a design effect of 5.7). Multilevel logistic regression models were used with dichotomous dependent variable (0 = non referral, 1 = referral) adjusted for clustering of patients (level 1) within practices (level 2) [33]. Initially, we fitted a baseline variance component or empty model (no independent variables) followed by the model with patient and practice variables (Model 1). ICC was calculated using the latent variable method. The (standard) logistic distribution has variance π^2^/3 = 3.29 and hence this can be taken as the level 1 variance. As both the level 1 and 2 variances are on the same scale, the following formula was used: ICC = (level 2 variance)/(level 2 variance + 3.29) [34]. All multi-level models were performed with MLwiN version 2.0. [33].

### Ethics

This study was approved by the University of New South Wales Human Research Ethics Committee. All participants provided written informed consent.

## Results

### Participants

**Figure 1:**
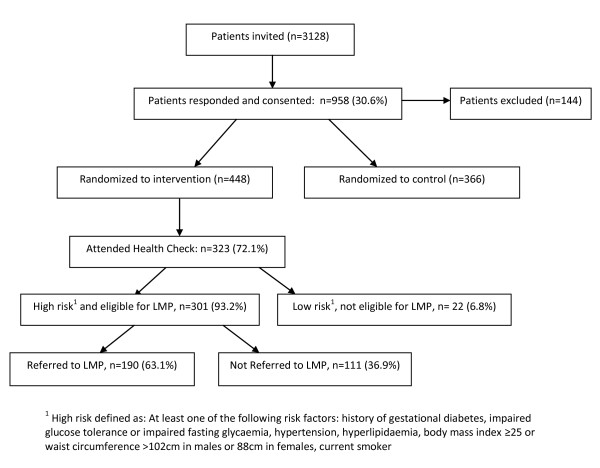
**Patient recruitment and referral to the HIPS Lifestyle Modification Program. **^1^High risk defined as: At least one of the following risk factors: history of gestational diabetes, impaired glucose tolerance or impaired fasting glycaemia, hypertension, hyperlipidaemia, body mass index ≥25 or waist circumference >102cm in males or 88cm in females, current smoker.

A total of 3128 patients were invited to participate in the HIPS trial, of whom 958 (30.6%) consented. From these, 144 were excluded as the GPs subsequently determined that they did not meet eligibility criteria, mostly due to existing diabetes or ischaemic heart disease (84 patients), or other serious disease making them unsuitable to participate. This left 814 patients (27.3% of total eligible). After randomization of practices there were 448 patients in the intervention group of whom 323 (72.1%) attended for a health check. Twenty two (6.8%) had no risk factors identified and were therefore not eligible for referral to the LMP. Of the 301 patients eligible for referral, 190 (63.1%) were referred and 111 (36.9%) were not referred to the LMP (Figure1).

### Characteristics of patients by referral status

Characteristics of patients by referral status are shown in Table1. Patients referred to the LMP were more likely to live in socio-economically disadvantaged areas, to have elevated BMI, and be physically inactive, with no difference in other demographic or risk factors. Referred patients were more likely to be in the contemplation, preparation or action stage of change for increasing fruit and vegetable intake, decreasing dietary fat, increasing physical activity and losing weight, than patients not referred. However there were no differences in stage of change for addressing alcohol or tobacco use. Those referred were also more likely to report having received advice from their GP for physical activity, but not nutrition, in the three month prior to the intervention.

**Table 1:** Characteristics of eligible patients referred compared to those not referred to the LMP (n = 301)

	**Referred to HIPS program (n = 190)**		**Not referred (n = 111)**		**Significance**
	**N**	**(%)**	**N**	**(%)**	**p-value**
**Demographic characteristics**					
Female	118	(62.1)	69	(62.2)	0.992
Age: 40–54 years	41	(21.6)	29	(26.1)	0.368
55-64 years	149	(78.4)	82	(73.9)	
SEIFA quintile:					
1 – poorest	26	(13.7)	8	(7.2)	**0.036**
2	16	(8.4)	4	(3.6)	
3	121	(63.7)	80	(72.1)	
4	10	(5.3)	2	(1.8)	
5 – richest	17	(8.9)	17	(15.3)	
Post-secondary qualification	89	(47.6)	52	(47.7)	0.985
Employment:					
Employed	124	(66.0)	75	(67.6)	0.918
Retired	30	(16.0)	18	(16.2)	
Not working (other reasons^1^)	34	(18.1)	18	(16.2)	
Accommodation:					
Owner-occupied	158	(84.0)	91	(82.7)	0.768
Rented or other	30	(16.0)	19	(17.3)	
Primarily speak English at home	165	(86.8)	90	(81.1)	0.180
**Patient health risk profile**					
Blood pressure (>140/90) or on treatment	18	(11.9)	9	(10.0)	0.647
Lipids (TG > 2.0, LDL > 2.5 or TC >4.5, or on treatment)	183	(97.3)	106	(96.4)	0.730
BMI ≥25	148	(78.7)	73	(67.6)	**0.034**
Diet risk	155	(81.6)	83	(74.8)	0.162
Physical inactivity	120	(63.5)	46	(42.2)	**<0.001**
Alcohol risk	59	(38.6)	33	(37.1)	0.819
Tobacco	19	(10.2)	11	(10.2)	0.993
Number of behavioural risk factors^1^:					
0	7	(3.7)	5	(4.5)	0.122
1	20	(10.5)	21	(18.9)	
2 or more	163	(85.8)	85	(76.6)	
Health status excellent/very good/good	159	(83.7)	97	(87.4)	0.385
**Patient personality/psychological factors**					
Psychological distress (K10score 16+)	70	(38.9)	32	(31.1)	0.187
Health information seeking behaviour:					
Doctor-Dependent Active	0	(0)	0	(0)	0.264
Doctor- Dependent Passive	64	(40)	43	(47.3)	
Independent Active	0	(0)	0	(0)	
Independent Passive	96	(60)	48	(52.7)	
**Patient readiness to change**					
Contemplation/preparation/action stage of change for increasing fruit and vegetables	91	49.5	47	43.9	**0.034**
Contemplation/preparation/action stage of change for decreasing dietary fat intake	107	58.5	51	48.1	**0.007**
Contemplation/preparation/action stage of change for increasing physical activity	131	70.1	61	58.1	**0.036**
Contemplation/preparation/action stage of change for losing weight	129	70.9	58	58	**0.011**
Contemplation/preparation/action stage of change for drinking less alcohol	57	43.8	36	46.2	0.913
Contemplation/preparation/action stage of change for quitting smoking	19	39.6	7	30.4	0.401
**Previous advice/referral for diet or physical activity**					
GP nutrition advice/referral in previous 3 months	65	(42.5)	33	(34.7)	0.225
GP physical activity advice/referral in previous 3 months	68	(44.4)	28	(30.4)	**0.030**

1 includes diet, BMI, physical activity, alcohol and tobacco risk.

Statistically significant p-values (<0.05) are shown in bold.

### Characteristics of practices by referral status

Patients referred to the LMP were more likely to be patients at smaller practices (1–3 GPs) than patients of larger practices. There were no other significant differences between practices (Table2).

**Table 2:** Practice characteristics for those referred versus those not referred (at Baseline)

	**Referred to HIPS program (n = 190)**		**Not referred (n = 111)**		**Significance**
	**N**	**(%)**	**N**	**(%)**	**p-value**
Rural location	148	(77.9)	76	(68.5)	0.071
Practice size:					
1–3 GPs	97	(51.1)	40	(36.0)	**0.012**
>3 GPs	93	(48.9)	71	(64.0)	
Practice nurse(s) work at the practice	128	(67.4)	66	(59.5)	0.167
Practice employs or rents rooms to allied health professionals	130	(68.4)	83	(74.8)	0.242
Practice has written preventive care protocols	131	(74.4)	86	(82.7)	0.110

Statistically significant p-values (<0.05) are shown in bold.

### Independent predictors of referral

In multilevel multivariate analysis, controlling for clustering by practice, factors independently associated with referral were elevated BMI (OR = 2.87; 95% CI = 1.10, 7.47), physical inactivity (OR = 2.90; 95% CI = 1.36, 6.14), being in contemplation, preparation or action stage of change for increasing physical activity (OR = 2.75; 95%CI = 1.07, 7.03), being rurally located (OR = 12.50; 95%CI = 1.43, 109.7) and being a patient of a small practice (1–3 GPs)(OR = 16.05; 95%CI = 2.74, 94.24) (Table3).

**Table 3:** Multi-level logistic regression models for referral to the LMP

**Explanatory variables**		**Empty model**	**Model 1**^**1**^
**Patient characteristics**		OR (95% CI)	OR (95% CI)
SEIFA quintile^2^	1 - poorest		1.00 (reference)
	2		9.87 (0.21, 471.1)
	3		1.44 (0.12, 17.53)
	4		3.36 (0.09, 122.4)
	5 - richest		7.18 (0.33, 155.1)
BMI	<25		1.00 (reference)
	≥25		**2.87 (1.10, 7.47)**
Physical activity	Active		1.00 (reference)
	Inactive		**2.90 (1.36, 6.14)**
Stage of change for increasing fruit and vegetables	Maintenance		1.00 (reference)
	Contemplation/preparation/action		0.70 (0.28, 1.78)
	Pre-contemplation		0.61 (0.11, 3.49)
Stage of change for decreasing dietary fat intake	Maintenance		1.00 (reference)
	Contemplation/preparation/action		1.56 (0.60, 4.05)
	Pre-contemplation		2.88 (0.47, 17.71)
Stage of change for increasing physical activity	Maintenance		1.00 (reference)
	Contemplation/preparation/action		**2.75 (1.07, 7.03)**
	Pre-contemplation		0.83 (0.14, 4.79)
Stage of change for losing weight	Maintenance		1.00 (reference)
	Contemplation/preparation/action		1.20 (0.41, 3.48)
	Pre-contemplation		1.59 (0.46, 5.52)
GP advice/referral for physical activity in previous 3 months	No		1.00 (reference)
	Yes		1.43 (0.68, 3.00)
**Practice characteristics**			
Practice location	urban		1.00 (reference)
	rural		**12.50 (1.43, 109.7)**
Practice size:	≥ 3 GPs		1.00 (reference)
	1-3 GPs		**16.05 (2.74, 94.24)**
Between patient variance (SE^3^)		1.176 (0.852)	1.045 (0.627)
Intra class correlation		0.263	0.241
Explained variance ^4^ (%)		-	11.14

*P < 0.05.

Statistically significant p-values (<0.05) are shown in bold.

Multilevel logistic regression^1^ Model 1: includes all variables found to be significant in univariate analysis. ^2^ 2006 index of relative socio-economic advantage/disadvantage, ^3^ Standard error, ^4^ Explained ‘between practice’ variance using the variance in the empty model as reference.

## Discussion

This paper has identified patient and practice predictors of referral to a lifestyle modification program among high risk patients participating in the intervention arm of a trial of prevention of CVD in primary care. Patient factors found to be independently associated with referral were elevated BMI, physical inactivity, and being in contemplation, preparation or action stage of change for increasing physical activity. Smaller and rurally located practices were significantly more likely to refer patients. Socio-economic status, readiness to change other lifestyle risks and previous advice or referral for physical activity, while significant in univariate analyses, were not significant in the multivariable analysis.

The HIPS program was successful in increasing the referral rate from less than 10 percent of high risk patients prior to the intervention[13] to over 60 percent with the intervention. The evidence-based and structured program, free of charge to patients and offered with coordination and support for referral to the program, appears to have overcome some of the previously identified access problems [14-17]. Moreover, referral patterns became more appropriate, with GPs more likely to refer patients who were overweight or obese, inactive and/or ready to change physical activity levels than other patients. The association with overweight or obesity is consistent with the findings at baseline[13] and supported by recent evidence[8,9]. The new associations with risk and readiness to change physical activity, which were not significant at baseline,[13] suggest that the general practice training and support provided had an effect in encouraging GPs to tailor interventions to patients’ level of risk and motivation.

Patients attending rural practices and small practices with 1–3 GPs were also more likely to be referred. Although the amount of variance explained at the practice level was small, rural practice and smaller practice size was positively associated with referral. The former was interesting given the generally longer travel distances to the HIPS programs in rural areas, and that baseline data from the participating GPs found that rural GPs had lower scores on lifestyle management of overweight and pre-diabetic patients[14]. The latter may have been due to patients in larger practices being offered “in-house” interventions. Additionally, GPs in smaller and rural practices may have had closer personal relationships with their patients, enhancing both their assessment of the patients readiness to address their lifestyle and their ability to motivate their patients to do so. Alternatively GPs from smaller practices and rural locations may have been more engaged with the project and thus made more effort to refer patients, or have previously experienced greater difficulties accessing suitable referral programs, and thus embraced the opportunity offered by the intervention with greater enthusiasm. Whatever the reason the implications for primary care organisations and referral programs is that smaller and rural practices may be more likely to refer if the barriers of cost and availability are addressed.

However, in spite of the measures taken to address access barriers, over one third (36.9%) of high risk, eligible patients were not referred to the LMP program. This may reflect an assessment by the GP that a particular patient was not sufficiently motivated to be likely to attend, consistent with the training provided on tailoring the program to patients’ stage of change. However, a substantial number of patients who were ready to change their behaviours were not referred. Previous research has identified GP perception of low patient motivation as a barrier to preventive care[35]. Vogt et al. (2010) identified two core dimensions in GPs perceptions of the effectiveness of medical interventions – the extent to which interventions involve patient effort and the size of the impact[36]. It is possible that some patients, despite being categorised as ready to make changes by the study questionnaire, were perceived by the GP as lacking motivation. This may lead to an assessment that the effort required by the patient is too great, particularly if an efficacious pharmaceutical intervention is available[37]. It is also possible that GPs did not consider the LMP particularly helpful in addressing smoking and alcohol consumption, as these risks were not associated with referral. Alternatively, other factors may have contributed to non-referral of some patients, including patient refusal, other illness limiting their ability to participate, or prioritisation of other issues at the time.

Analysis of attendance rates at the LMP revealed that over a third of the patients who were referred (36.5%) did not attend [38]. Attendance rates were mainly related to external factors including participants work commitments and poor physical access to the program[38], while referral was related to individual’s health risk status and motivation to change. This suggests that different factors influence referral and attendance rates, with referral driven more by provider perceptions and attendance mediated by patient circumstance. Unfortunately, there is a risk that non-attendance may provide negative feedback to GPs – discouraging them from making future referrals. Highlighting that non-attendance is mainly related to external factors impacting on patients, rather than a reflection on the GPs may be important to include in future training for GPs.

A limitation of the study is that the trial was primarily designed to assess the impact of the intervention on patient outcomes rather than to evaluate referral patterns. Consequently, the sample size is relatively small, which may have limited our ability to detect some important patterns in referral. The generalisability of the findings are also limited – only 31% of patients responded to the initial invitation to participate, and only 72% of those consenting attended for their health check. Thus, the majority of patients who might have potentially benefited from the intervention did not participate. Those who did participate may not be representative of the 45 to 64 year old Australian population – while over 80% lived in owner-occupied homes, which is comparable to 78.7% of this age group for Australia as a whole[39], nearly 48% had post-secondary qualifications which is considerably higher than the general population in this age group, of whom only 40.6% have post-secondary qualifications [40]. While it is impossible to determine the extent to which the participants differ from non-participants or the reasons for non-participation, it is likely that non-participants either did not perceive themselves to be at risk, or did not perceive sufficient benefit from participation. We were also not able to link patients to individual GPs, but only to practices which may involve several GPs. This prevented us from assessing the impact of individual GP characteristics on referrals. Finally, the study only explores referral patterns to a LMP provided as part of a trial, with considerable support for referral, and does not reflect referral patterns in current ‘real world’ situations where these supports are not available. Rather it reflects what might be possible if such programs were provided through primary care organisations.

## Conclusions

Providing a well-structured evidence-based lifestyle intervention, free of charge to patients, with coordination and support for referral processes resulted in over 60% of participating high risk patients being referred for disease prevention. Contrary to expectations, referrals were more frequent from rural and smaller practices suggesting that these practices may be more ready to engage with these programs

## Competing interests

The authors declare that they have no competing interests.

## Author contributions

All authors contributed to the conception and design of the study and interpretation of the data. RL, MP and UJ contributed to data analysis, and UJ undertook the multilevel analysis. MP drafted the initial manuscript. All authors contributed to revising the manuscript and approved the final version.

## Pre-publication history

The pre-publication history for this paper can be accessed here:

http://www.biomedcentral.com/1472-6963/12/234/prepub
